# Apertureless scanning near-field optical microscopy of sparsely labeled tobacco mosaic viruses and the intermediate filament desmin

**DOI:** 10.3762/bjnano.4.60

**Published:** 2013-09-11

**Authors:** Alexander Harder, Mareike Dieding, Volker Walhorn, Sven Degenhard, Andreas Brodehl, Christina Wege, Hendrik Milting, Dario Anselmetti

**Affiliations:** 1 Experimental Biophysics and Applied Nanoscience, Faculty of Physics and Bielefeld Institute for Biophysics and Nanoscience (BINAS), Bielefeld University, Universitätsstrasse 25, D-33615 Bielefeld, Germany; 2 Department of Molecular Biology and Plant Virology, Institute of Biology, University of Stuttgart, Pfaffenwaldring 57, D-70569 Stuttgart, Germany; 3 Erich and Hanna Klessmann Institute for Cardiovascular Research & Development (EHKI), Heart and Diabetes Center NRW, Ruhr University Bochum, Georgstraße 11, D-32545 Bad Oeynhausen, Germany; 4 Libin Cardiovascular Institute of Alberta, Department of Cardiac Sciences, University of Calgary, 3280 Hospital Drive NW, T2N4Z6, AB, Canada

**Keywords:** apertureless scanning near-field optical microscope, atomic force microscopy, fluorescence microscopy

## Abstract

Both fluorescence imaging and atomic force microscopy (AFM) are highly versatile and extensively used in applications ranging from nanotechnology to life sciences. In fluorescence microscopy luminescent dyes serve as position markers. Moreover, they can be used as active reporters of their local vicinity. The dipolar coupling of the tip with the incident light and the fluorophore give rise to a local field and fluorescence enhancement. AFM topographic imaging allows for resolutions down to the atomic scale. It can be operated in vacuum, under ambient conditions and in liquids. This makes it ideal for the investigation of a wide range of different samples. Furthermore an illuminated AFM cantilever tip apex exposes strongly confined non-propagating electromagnetic fields that can serve as a coupling agent for single dye molecules. Thus, combining both techniques by means of apertureless scanning near-field optical microscopy (aSNOM) enables concurrent high resolution topography and fluorescence imaging. Commonly, among the various (apertureless) SNOM approaches metallic or metallized probes are used. Here, we report on our custom-built aSNOM setup, which uses commercially available monolithic silicon AFM cantilevers. The field enhancement confined to the tip apex facilitates an optical resolution down to 20 nm. Furthermore, the use of standard mass-produced AFM cantilevers spares elaborate probe production or modification processes. We investigated tobacco mosaic viruses and the intermediate filament protein desmin. Both are mixed complexes of building blocks, which are fluorescently labeled to a low degree. The simultaneous recording of topography and fluorescence data allows for the exact localization of distinct building blocks within the superordinate structures.

## Introduction

Scanning near-field optical microscopy (SNOM) provides sub-wavelength optical resolution [1]. The sample is excited by the strongly confined near-field at the tip apex, which is induced by the dipolar coupling between the incident light and the probe. Moreover, coupling between the fluorophore dipole and the tip can lead to both, fluorescence enhancement and quenching. In summary, all these effects highly depend on several experimental parameters such as the distance between tip and fluorophore, the probe geometry and material and the polarization of the incident light. Furthermore, the distance-control feedback loop of the probe can be used to gain topographical information as it is done in atomic force microscopy (AFM). Thus, SNOM generally allows the acquisition of both optical and topographical information. Various conceptual approaches have been reported: In fiber SNOM the sample is illuminated through the aperture of a metal coated optical fiber tip [2]. Due to the blunt tip this method shows low topographic resolution. Besides, the optical resolution can be increased by the diminution of the tip aperture, which chokes the optical throughput. Therefore, apertureless SNOM probes appear favorable [3–7]. Commonly, metallic and metallized probes expose a strong field enhancement and dipolar coupling between fluorophore and tip, which result in a remarkable increase of the observable fluorescence emission. However, the interaction between dye and tip apex can be manifold and depend crucially on the experimental setup and the sample. As a result the fluorescence emission rate can be both significantly enhanced or reduced at distances up to some 10 nm [6,8–11], and single-molecule images can show complex fluorescence patterns [3,12–13]. Silicon probes expose only a moderate field enhancement and the dipolar coupling between probe and dye is less pronounced [4]. Even though, silicon tips can quench the fluorescence emission at close proximity [14]. Moreover, the elaborate probe design of custom-made tips demand complex fabrication processes and substantial experience [3,7,12–13,15–17]. Here, we report on an aSNOM approach using commercially available monolithic silicon AFM cantilevers. These expose a sharp tip apex for high topographic resolution and strong field confinement. Additionally, the field enhancement is sufficient for an adequate signal to noise ratio (SNR). Besides, fluorescence and topography data are inherently aligned allowing easy superposition and localization of single fluorescence peaks within topographic features. Many biological systems from single molecules to cells and viruses are mixed complexes that are composed of specific building blocks. Their structure and function directly depend on the exact composition of all constituents. By using two exemplary systems we demonstrate that aSNOM enables the precise localization of single fluorescently labeled components within these structures.

**Figure 1:**
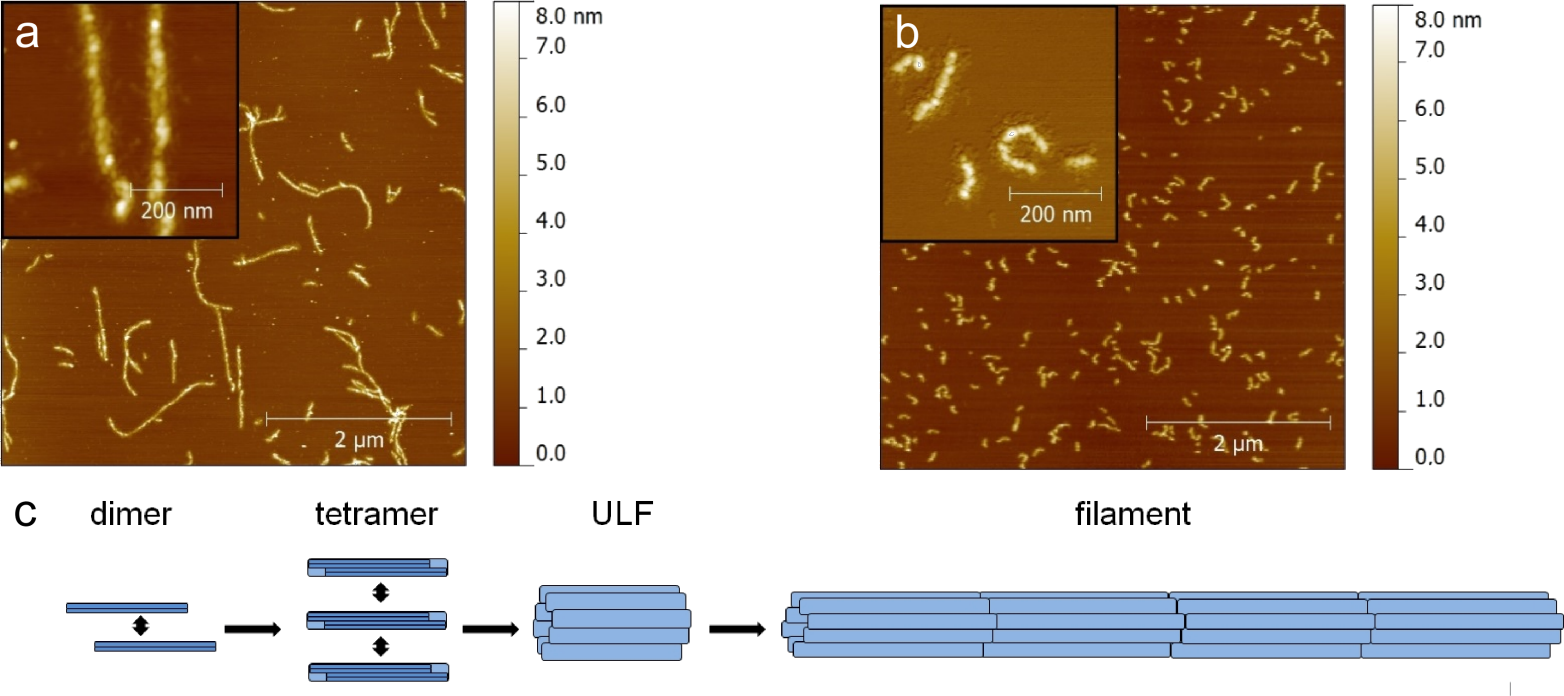
AFM topography scans of desmin filaments under ambient conditions. a) Filaments assembled from wild-type proteins are several micrometers long and expose a vague right handed twist. b) Filaments assembled from mutated desmin protein (deletion mutation p.E114del, for details see [22]) are significantly shorter and show a completely different surface morphology. c) Assembly model of type II intermediate filaments [24]. Desmin monomers associate to parallel coiled coil dimers. Dimers form antiparallel tetramers which assemble to unit length filaments (ULF). Filaments elongate by longitudinal assembly of ULF.

The desmin intermediate filament protein assembles to extensive fibrous networks, which are an integral part of the cytoskeleton of heart muscle cells. Several mutations of the desmin gene are associated with severe muscle diseases like arrhythmogenic right ventricular cardiomyopathy (ARVC) [18–22]. ARVC leads to cardiac arrhythmia and a degeneration of the heart muscle predominantly of the right ventricle. It has been found recently that the desmin filament assembly can be heavily affected by mutations (Figure 1) [23]. Nevertheless, the molecular patho-mechanism leading to a degeneration of the heart muscle is still unknown. Of note, desmin mutations follow a dominant inheritance, which means that only one of the two alleles carries the mutation. Consequently, both mutant and wild-type desmin forms are present in the same cell. Moreover, it has been shown recently by means of dual color photo activation localization microscopy (PALM) that both protein species coexist within the same filaments [23]. However, the distribution of both protein types in the early stage of the desmin filament formation, i.e., oligomers and unit length filaments (ULF), respectively, is still not known and might give insight in the patho-mechanism.

The tobacco mosaic virus (TMV) is a tubular shaped plant virus with a length of 300 nm and a diameter of 18 nm. It is composed of 2130 identical capsid protein subunits helically arranged on a single RNA strand thereby enclosing an inner longitudinal channel [25]. Apart from plant research, TMV is also important for nanotechnology applications of virus-derived biotemplates [26–29]. The self-assembly of the capsid components and the homogeneous nucleoprotein tube diameter make TMV an attractive scaffold for nanotechnological applications such as the formation of TMV-based semiconductive ZnO composites in field effect transistors [30]. Modifications of the viral shell proteins allow the introduction of target-oriented chemical functionalities on the nanotube surface [31]. By means of fluorescent labeling these modifications can now easily be localized by the use of aSNOM.

## Results and Discussion

**Figure 2:**
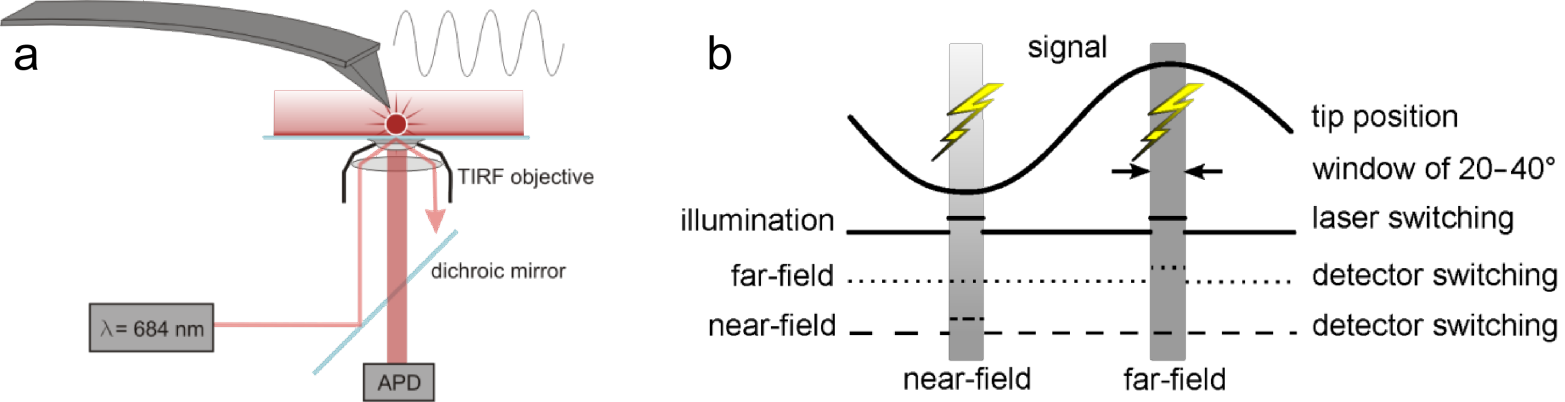
a) Scheme of the experimental setup. b) The sample illumination and fluorescence readout is correlated with the cantilever position. The far-field readout is triggered at the upper reversal point of the cantilever whereas the near-field data is recorded at the lower reversal point.

We used a home-built aSNOM setup that consists of an AFM head mounted on a discretely designed inverted optical microscope (Figure 2a). To center the cantilever tip in the confocal detection volume the AFM is positioned on a separate 2D linear stage. The sample holder is attached on a 3D piezo stage (P-733.3, Physik Instrumente, Karlsruhe, Germany) for lateral sample scanning and vertical fine alignment. The AFM head holds a separate piezo actuator (PSt 150/2x3/5, Piezomechanik, München, Germany) for vibrational excitation of the AFM cantilever and surface distance control. The system is controlled by a commercially available scanning probe microscopy control system (Nanonis OC4, Specs, Zürich, Switzerland). The sample is evanescently illuminated by a laser diode (RLT6830MG, λ = 685 nm, 30mW, Roithner Lasertechnik, Vienna, Austria) through a high numerical aperture (NA) microscope objective (CFI Apochromat 100× TIRF, NA = 1.49, Nikon, Tokyo, Japan). The laser diode is linearly polarized and directed to the sample such that the polarization is parallel to the plane of incidence (p-polarization). Thus, the component of the wave vector perpendicular to the sample surface is enhanced. For detection of the fluorescence emission we used a confocal scheme with a gated avalanche photodiode (APD) (SPCMAQR-13, Perkin Elmer, Waltham, MA, USA). Notable, the sample illumination and fluorescence detection is synchronized with the cantilever oscillation in such a way that we gain two fluorescence images corresponding to both reversal points of the cantilever oscillation (Figure 2b). The gating window corresponds to a phase width of 20–40°.

**Figure 3:**
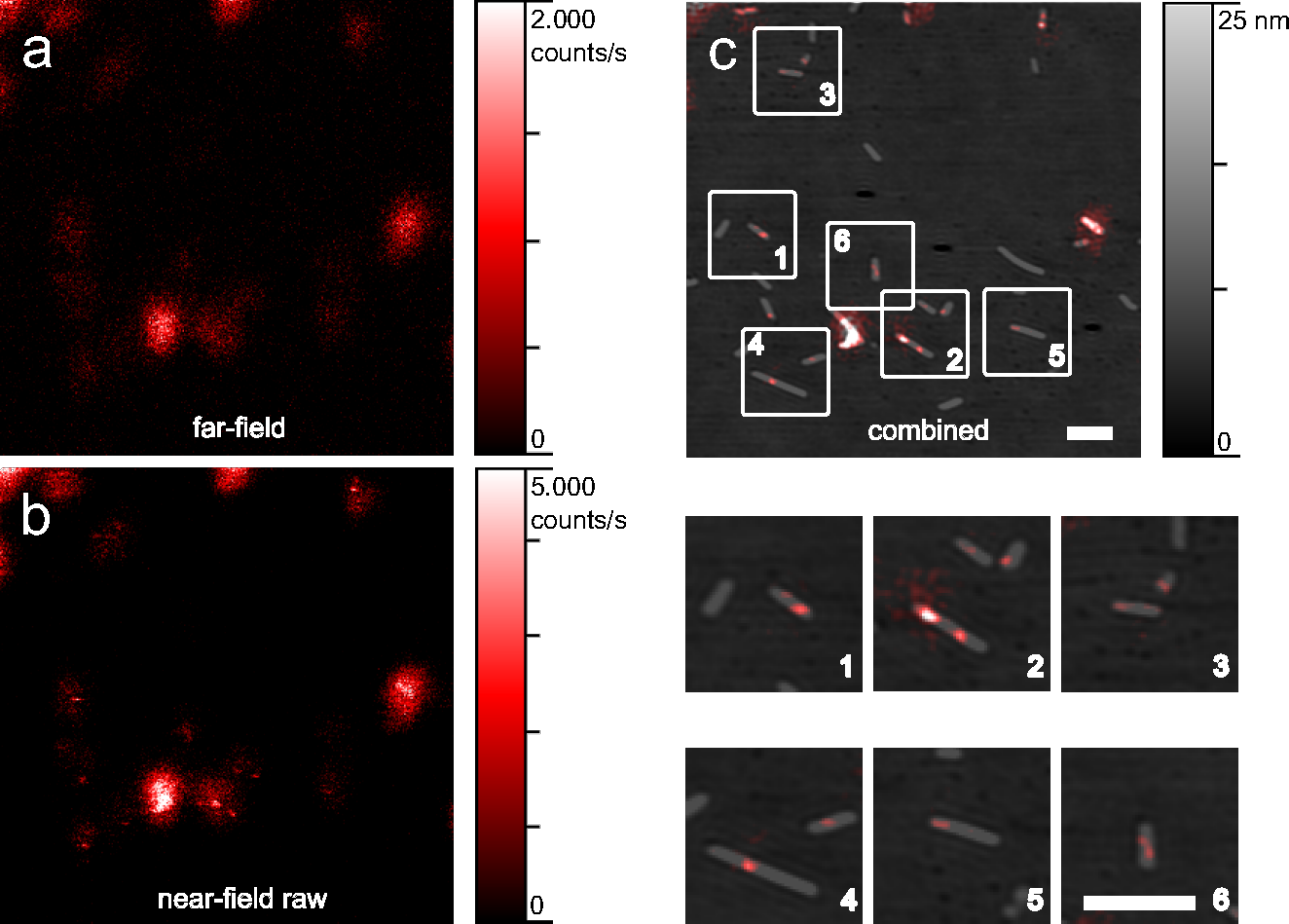
aSNOM dataset of TMV (a–c, 5×5 µm^2^). a) Blurry far-field fluorescence acquired at the upper reversal point of the oscillating cantilever. b) The raw near-field fluorescence was recorded at the lower reversal point of the cantilever. The near-field contribution is located at the center of the dim stray fluorescence. c) The plain near-field fluorescence data is superimposed on the AFM topography. The marked areas are magnified for better visibility. All scale bars indicate 500 nm.

Modified TMV with sparsely distributed fluorescent dyes, which label selectively addressable groups, were immobilized as described and investigated. A single aSNOM scan yields three different data sets: AFM topography, far-field (Figure 3a) and (raw) near-field fluorescence (Figure 3b) data. The unprocessed near-field fluorescence image still contains a significant far-field background. When a single dye molecule is moved through the focal detection volume which has a characteristic radius of approx. 300 nm, the fluorescence emission is only enhanced at dye–tip distances lower than approx. 10 nm. Consequently, the raw near-field data exposes stray far-field fluorescence. During post-processing the far-field image is subtracted from the near-field image. The result is subsequently low-pass filtered as described. Even though silicon probes can also quench the fluorescence emission at close proximity, we did not detect the same. This is most probably due to the fact that the detector count rate is integrated over a comparably broad phase window of 20–40°. Topography and processed near-field fluorescence can then be superimposed without further lateral adjustment (Figure 3c). The combined data allow to easily determine the degree of modification and the loci of anchor groups equipped with dye molecules.

**Figure 4:**
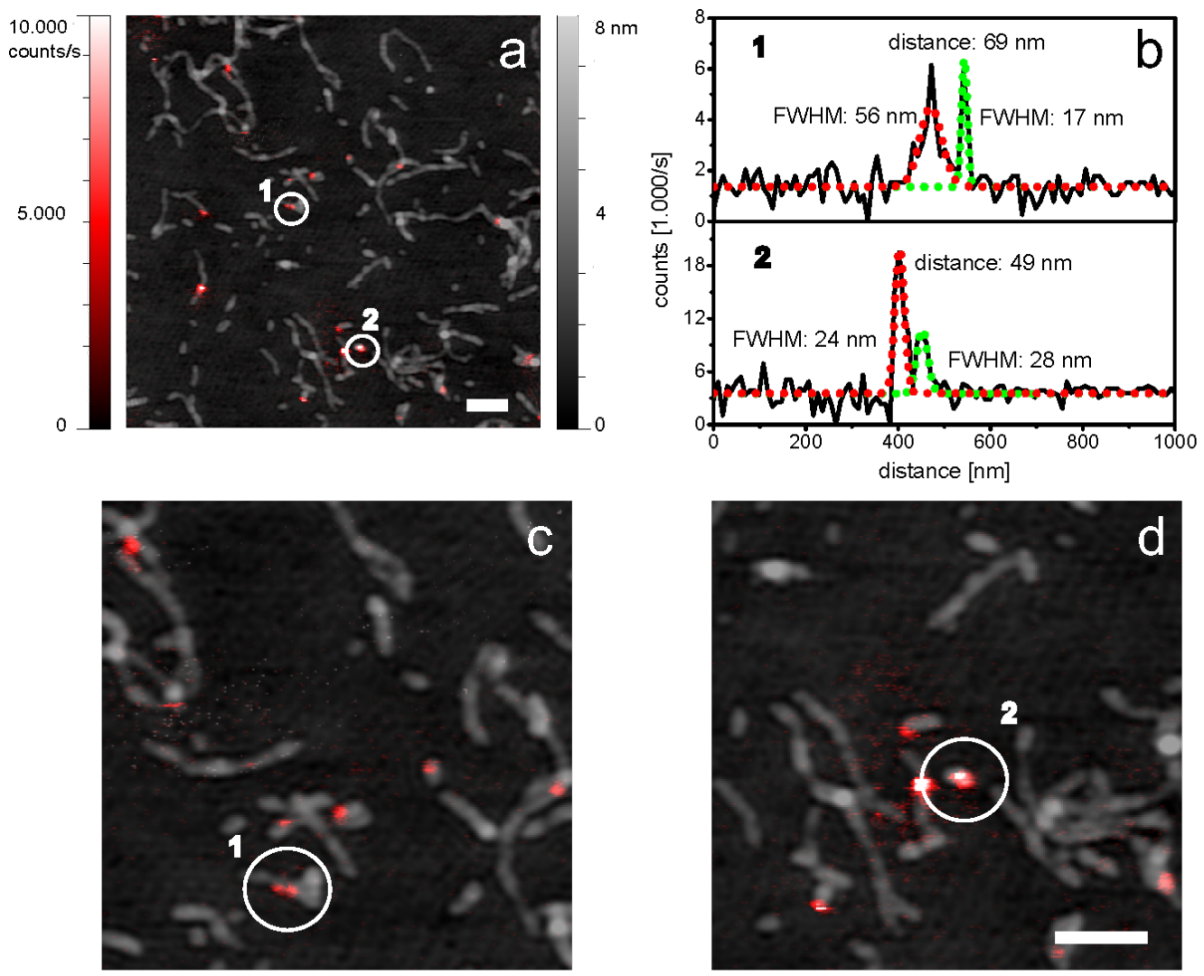
aSnom data of fluorescently labeled desmin filaments. a) Combined fluorescence and topography dataset of wild type desmin. b) Fluorescence intensity cross sections of two adjacent fluorophores are extracted to estimate the optical resolution. The peaks are approximated by a Gaussian distribution to determine the FWHM. c,d) The marked areas are magnified for better visibility. All scale bars indicate 500 nm.

**Figure 5:**
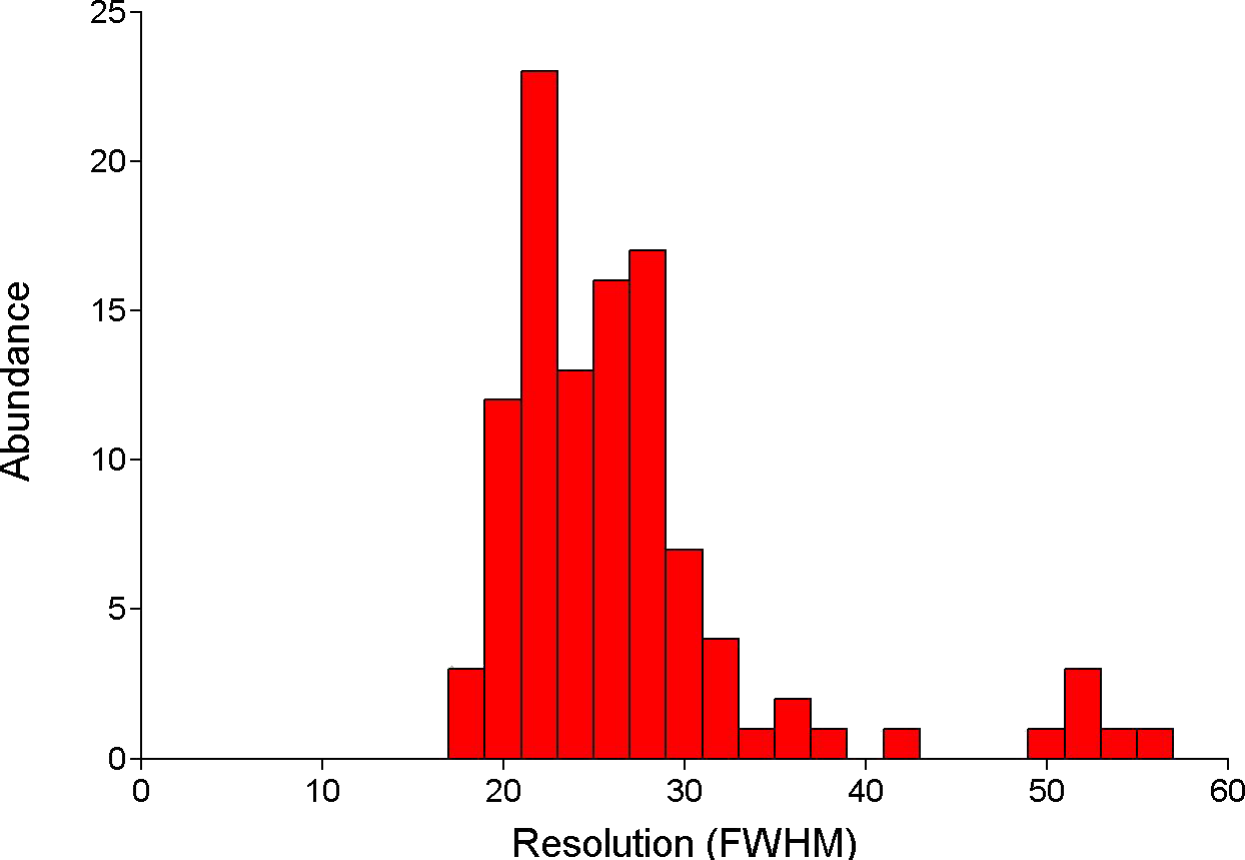
Histogram of the FWHM of fluorescence peaks obtained from labeled desmin filaments.

Similar to the TMV capsid, desmin is a construct of (quasi-) identical building blocks, which assemble to a complex higher-level structure. We prepared sparsely labeled wild type desmin and immobilized the filaments as described. The topography data shows fibrous structures as it is known from unlabeled desmin filaments. The labeling procedure was derived from a protocol for the homologue intermediate filament vimentin [32]. Even though fluorescence labeling can disturb the directed association of proteins due to steric hindrance, the filament assembly was apparently not inhibited. The merged dataset exposes filaments with sharp and clearly detectable sparsely distributed fluorophores. Even in the case of fluorescence signals, which were detected adjacently, we observed distances in the range of 50–70 nm, which coincide very well with the length of ULF (about 60 nm, Figure 4) [33]. Consequently, the labeled monomers are most likely located in vicinal ULF. The optical resolution was estimated by means of approximating a Gaussian distribution to the fluorescence peaks. We found single fluorophores with a full width at half maximum (FWHM) down to 17 nm. The average FWHM was 25 nm (Figure 5). Moreover, the optical SNR was better than 10 for all analyzed dyes, which corresponds nicely to recently published results [5]. It is noteworthy that the resolution for individual fluorophores can differ significantly (Figures 3, 4 and 5) from the average. This is most probably due to the comparably high corrugation and complexity of TMV and desmin, which can limit the accessibility of individual dyes. The topographic resolution was found to be lower compared to conventional AFM, since the morphology of the filaments could not be resolved. Moreover, the filaments appeared slightly broader (data not shown). This still seems to be a conceptual issue of combined optical and scanning probe microscopy: The sample has to be accessible from top and bottom. Furthermore, high numerical aperture objectives constrict to the use of thin glass cover slips with a thickness of 150–170 µm as sample substrates. Consequently, the setup, as well as the substrates, is more susceptible to vibrational noise. Nevertheless, aSNOM has shown to be an ideal tool for the localization of single building blocks within superordinate structures.

## Conclusion

We demonstrated that aSNOM is an appropriate tool to simultaneously image topography and fluorescence of complex higher level structures down to the single-molecule level. The achieved resolution allows the allocation of single molecular building blocks within these constructs. In the case of the intermediate filament desmin this enables a detailed analysis of the filament assembly mechanism to explore the molecular patho-mechanism. Furthermore, the use of unmodified, commercially available standard AFM cantilevers is timesaving and cost-effective compared to other elaborate near-field probes. Since the tip apex serves as probe for topography and fluorescence the datasets are intrinsically aligned. Consequently, image processing is very easy and the data can be superimposed without further adjustment. Both, the AFM and the fluorescence part of the setup are highly versatile: For example the extension to dual color fluorescence is possible without major modifications. Generally, the setup also allows imaging in liquid media, which makes it predestined for biological samples. Furthermore, the intermittent fluorophore excitation reduces photo-bleaching and thus extends imaging time.

## Experimental

### Image processing

The unprocessed near-field image contains a significant amount of parasitic far-field fluorescence. By subtracting the background far-field fluorescence signal from the near-field fluorescence signal and applying an additionally line-by-line low-pass filter to the subtraction in order to reduce artificial noise, we generated a corrected near-field image with considerably reduced far-field artifacts. Of note, the low-pass filter primarily smoothes individual “noisy pixels” and hardly alters the shape or the width of the fluorescence peaks. The resulting high resolution fluorescence data are then superimposed on the topography. All image processing is done with the SPIP image processing software (Image Metrology A/S, Hørsholm, Denmark). All SNOM experiments were done with monolithic silicon cantilevers (ATEC-NC, Nanosensors, Neuchatel, Switzerland). The tetrahedral tip protruding from the end of the lever ensures visibility of the tip apex and consequently facilitates the alignment relative to the illumination laser. As fluorescent dye we used maleimide-modified Atto740 (Atto-Tec, Siegen, Germany) which has a remarkable photo-stability and a preferably low quantum yield of 10%. Low quantum efficiencies are beneficial as dipole–dipole coupling effects between the cantilever tip and dye increase the radiative decay rate and thus enhance the quantum yield [34].

### Sample preparation

The desmin protein was expressed in *E.coli*, isolated and purified as reported recently [20]. Desmin monomers expose a single cysteine, which is an ideal anchor for dye attachment. The labeling procedure is derived from the protocol which has recently been published for the homologue IF vimentin [32]. Briefly: desmin is dialyzed for one hour at room temperature against labeling buffer (5 M urea, 5 mM Tris-HCl , pH 7.0–7.5). Atto740 is solubilized in water-free DMSO to 5 mg/mL and added in a 10:1 molar excess. After 30 minutes the unbound dye is blocked by the addition of cysteamine (1 M in H_2_O to a concentration of 100 mM and incubation for 1 hour at room temperature). The labeled desmin is separated from free dye molecules by dialysis (against 8 M urea, 5 mM Tris-HCl, 1 mM DTT pH 8.4) and stored at −8 °C. The degree of labeling (DOL) is determined by absorption spectra to DOL = 2.2. Labeled and unlabeled desmin are mixed in a 1:10 molar ratio and stepwise dialysed against 5 mM Tris-HCl, 1 mM DTT at pH 8.4. Assembly is initialized by addition of an equal volume of assembly buffer (200 mM NaCl, 45 mM Tris-HCl, pH 7) to a desmin solution with a concentration of 0.55g/L and incubated for one hour at 37 °C.

TMV particles are isolated from systemically infected *Nicotiana tabacum* 'Samsun' nn plants according to Gooding and Hebert [35]. A TMV mutant, which presents a single thiol-group on every capsid protein subunit of the virus surface (TMV_Cys_, 2130 coupling sites per viral nanotube), is used [31]. The labeling procedure is performed with a substoichiometric molar ratio of 0.02:1 of Atto740 maleimide (Sigma-Aldrich, München, Germany) to viral capsid protein, in sodium potassium phosphate (SPP) buffer (10 mM SPP buffer, pH 7.2), with a virus concentration of 1 µg/µL in a total volume of 100 µL. The reaction mixture is incubated for 9 h at 30 °C in the dark, gently shaken at 300 rpm (Thermomixer Compact, Eppendorf, Hamburg, Germany). An amount of 300 µL SPP buffer is added and the unbound dye is removed by ultracentrifugation for 2 h at 15 °C and 120,000*g* (Sorvall Ultra Pro 80, Thermo Fisher Scientific Inc., Waltham, USA). The pellet containing the modified virions is resuspended in 100 µL SPP buffer and the degree of labeling is determined by absorption spectra (NanoDrop ND-1000, PEQLAB, Erlangen, Germany) to DOL = 1.8%.

Fluorophore-exposing desmin filaments (130 ng/µL) and TMV_Cys_ (1 ng/µL) are immobilized onto etched and cleaned glass slides (Menzel Gläser, Germany) as follows: The slides are sonicated for 20 min in acetone p. a. (Sigma, Germany) and thoroughly rinsed with ethanol p. a. (Sigma, Germany) and Milli-Q water (18.2 MΩ·cm; Millipore, Germany). The cleaned glass slides are additionally etched with 65% nitric acid p. a. (Roth, Germany) for 2 min to activate the glass surface. Subsequently the glass slides are washed with Milli-Q water and dried in a gentle nitrogen stream. Desmin and TMV, respectively, are pipetted on the slides to incubate for several seconds. Unbound desmin or TMV are rinsed from the slides with Milli-Q water. Finally the samples are dried in a nitrogen stream.
